# Comparative analysis of multifaceted neural effects associated with varying endogenous cognitive load

**DOI:** 10.1038/s42003-023-05168-4

**Published:** 2023-07-31

**Authors:** Leisi Pei, Georg Northoff, Guang Ouyang

**Affiliations:** 1 Faculty of Education, The University of Hong Kong, Hong Kong, China; 2 Institute of Mental Health Research, Mind, Brain Imaging and Neuroethics Research Unit, University of Ottawa, Ottawa, Canada

**Keywords:** Cognitive neuroscience, Neurophysiology

## Abstract

Contemporary neuroscience has firmly established that mental state variation concurs with changes in neural dynamic activity in a complex way that a one-to-one mapping cannot describe. To explore the scenario of the multifaceted changes in neural dynamics associated with simple mental state variation, we took cognitive load – a common cognitive manipulation in psychology – as a venue to characterize how multiple neural dynamic features are simultaneously altered by the manipulation and how their sensitivity differs. Electroencephalogram was collected from 152 participants performing stimulus-free tasks with different demands. The results show that task demand alters wide-ranging neural dynamic features, including band-specific oscillations across broad frequency bands, scale-free dynamics, and cross-frequency phase-amplitude coupling. The scale-free dynamics outperformed others in indexing cognitive load variation. This study demonstrates a complex relationship between cognitive dynamics and neural dynamics, which points to a necessity to integrate multifaceted neural dynamic features when studying mind-brain relationship in the future.

## Abstract (web summary)

Analysis of EEG data from 152 participants performing stimulus-free tasks with different demands suggests that manipulation of cognitive load by changing the task demand alters wide-ranging neural dynamic features.

## Introduction

Cognitive neuroscience research has firmly established that variations in mental state concur with changes in neural dynamics^1–7^. This has led to a surge of research work that aims to identify specific neural dynamic features (e.g., band-specific power) associated with complex cognitive variables, such as cognitive load and emotional states^8,9^. However, it is not a trivial task to pinpoint an isolated change in the neural dynamics responsible for a specific mental state variation due to the complexity of neural dynamic systems. For one, the variation of a specific mental state likely involves multiple sub-processes, each of which may change the neural dynamics in a different aspect. For another, the neural dynamic system is an active system with multiple interplaying functional modules that are constantly interacting with each other^10^. Change in one facet may lead to changes in others.

To demonstrate the multitude of neural dynamic changes engendered by mental state variations and comparatively analyze them, we adopted the concept of cognitive load in the present experimental investigation. Cognitive load has been a major venue via which applied neuroscience researchers have attempted to exploit neural dynamic signals for real-life use, e.g., improving teaching and learning, and enhancing work safety^8,11^. Despite variations in its definition across different fields^12–14^, its associated cognitive effect has been fairly agreed upon: increased cognitive load is associated with a subjective feeling of intensified mental activity and effort. From a neural computational point of view, increased cognitive load is associated with increased demand for neural resources and energy related to the information processing and computation for solving a task^15,16^. Implementation-wise, manipulation of the cognitive load has been mainly based on task difficulty. However, the construct of cognitive load defined by perceived mental intensity may not always be compatible with cognitive science approaches that are oriented to decoding fundamental and elementary cognitive processes: variation of cognitive load can be very heterogeneous such that it could implicate sensory processes, task modality, attention, emotion, and others. As such, it may not be meaningful to set cognitive load as the primary research object due to its high heterogeneity nature. There have been theories from the instructional design that taxonomize cognitive load into different sub-types, such as intrinsic, extraneous, and germane cognitive load^14^. However, such taxonomy is more oriented to learning outcomes and thus has inherent conceptual unclarity in terms of the underlying cognitive processes or activities it refers to^17–19^.

The present work is not positioned to clarify different types of cognitive load but rather aims to present the characteristics of various neural dynamic changes associated with a generic sense of cognitive load variation associated with task difficulty or complexity. To best avoid heterogeneity issues, we adopted tasks that are free of sensory processing, i.e., the tasks will be exclusively tapping into internally generated task processing activities associated with different task difficulties, thus different cognitive loads. Such cognitive load may be described as intrinsic cognitive load, but its meaning in the current context is fundamentally different from the intrinsic cognitive load in Sweller’s cognitive load theory^14^. To avoid this confusion, we will term it endogenous cognitive load in the present article, which refers to the workload required for performing a task that only requires processing internally generated information (e.g., silent counting). And we will comparatively characterize the multifaceted neural dynamics changes associated with this cognitive variable.

In terms of neural effects, the changes in neural dynamic features associated with cognitive load variation have been extensively demonstrated, but the vast majority of them only reported changes in a specific feature. Band-specific oscillation change is the most frequently reported one^20–27^. Briefly, theta oscillation (3–7 Hz) is enhanced with increasing cognitive load^28–30^, which has been attributed to the unique role of theta (especially the frontal midline theta) in general cognitive control and working memory operations^31–34^. Conversely, alpha oscillation (8–12 Hz) is attenuated with increasing cognitive load^8,30,35^. This may be linked to a broader phenomenon that alpha oscillation is suppressed during nearly all kinds of task states and is rebounded during idle (mentally unengaged) state^36,37^. The association between beta oscillation (13–30 Hz) and cognitive load variation remains nebulous – some studies reported increased beta oscillation with increasing cognitive load^38,39^ while others reported decreased beta oscillation^40–43^. Gamma oscillation has also been extensively found to be increased in general task engagement^44,45^ in a way that is correlated with cognitive load^26,46,47^ but see the opposite effect in default mode network^48^, A common neural account of this effect is that neural firing activity is intensified during task processing, which is mainly manifested in the gamma band^49–51^. Finally, in addition to separate oscillations, the effects of cognitive load have also been extended to the interaction between oscillations in different frequency bands, i.e., cross-frequency coupling^52^ with phase-amplitude coupling (PAC) being the predominant type. A common pattern is that the strength of PAC is enhanced by cognitive load^53,54^. This relationship can be understood from the proposed role that PAC plays in cross-region communication in large-scale brain networks^52^, which makes PAC an important neural dynamic metric to examine in cognitive load research.

In addition to the band-specific oscillations, the scale-free dynamics is another important facet of the temporal dynamics of neurophysiological activity^55,56^ that has also been found to be associated with cognitive load. The general pattern is that both task engagement and load increase modulate the scale-free pattern by rotating the frequency spectrum of brain activity counterclockwise^57–59^. This pattern is in rough agreement with decreased alpha and increased gamma power reviewed above. Such overlapping of effects implies the need to disentangle them when studying the mind-brain relationship^58,60^, which will also be studied in this work through the variable of cognitive load.

Together, our brief literature review above shows a considerable number of studies that have reported associations between cognitive load and neural dynamic features. However, they mostly reported on a specific facet of dynamic features (which differs across studies). According to our hypothesis, we aim to show the wide-ranging effects of cognitive load variation that permeate different frequency bands and henceforth, various facets of neural dynamics. This aim may be better achieved by a large sample size. In this study, we conducted a systematic investigation of the changes in multifaceted neural dynamics associated with variation of endogenous cognitive load based on a large sample (*n* = 152). To exclude confounding of sensorimotor processes, we administered tasks that engendered long periods of stimulus-free task states with different levels of internally imposed cognitive load. Specifically, the participants were instructed to perform three stimulus-free tasks, (1) sitting still and resting, (2) backward counting in an easy mode, (3) backward counting in a hard mode. We then analyzed the effects of the endogenous cognitive load variation on multiple neural dynamic features, including canonical band-specific oscillations, scale-free dynamics, cross-frequency phase-amplitude coupling, and their correlation with behavioral performance. These large sample size-based neural dynamics analyses revealed an informative picture of the relationships between the neurophysiological system and the mental system.

## Results

### Behavioral data related to task state difference

The average counting frequency (number of counting per second) is 1.16 (SD 0.41) Hz for the easy counting task and 0.23 (SD 0.10) Hz for the hard counting task. They are significantly different (paired *t*-test: *t*(151) = 27.95, *p* < 0.001). In the hard counting task, 96 (63.16%) participants arrived at a correct result, and 56 (36.84%) participants arrived at an incorrect result, which reflects the difficulty of the hard counting task. Concerning the possibility that the participants reporting an incorrect answer may not genuinely perform the task, in the subsequent neural results analysis, we also examined the robustness of the key results across these two groups of participants.

### Descriptive spectrum features across task states

**Fig. 1:**
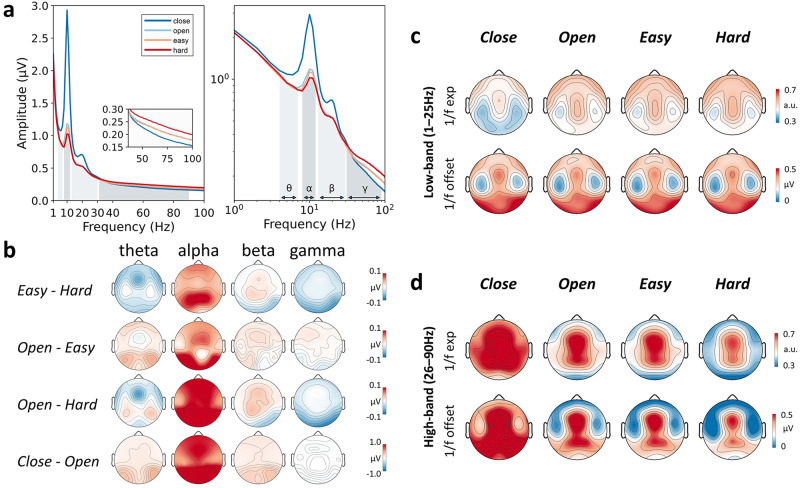
Variations in EEG dynamics across task states. **a** Grand average spectra for the four task states in both linear (left) and log-log (right) scales (averaged across all electrodes). Theta (4–7 Hz), alpha (8–12 Hz), beta (13–30 Hz), and gamma (31–90 Hz) bands were indicated in gray shades. **b** Grand average spatial distribution of the between-state difference in oscillation amplitude for the four canonical frequency bands. **c**, **d** Grand average spatial distribution of the 1/*f* exponent (noted as exp) and offset parameters fitted from 1–25 Hz (**c**) and from 26–90 Hz (**d**) for each task state. Note: a.u. refers to arbitrary unit.

To obtain a straightforward description of the cognitive load-related differences in the EEG pattern, we first examined the spectrum features. The frequency amplitude spectrum is visualized for each task state in both linear and log-log spaces (Fig. 1a). Visually, there appears to be a systematic variation in the spectrum pattern as a function of the four task states. The two most conspicuous effects are: task states with higher cognitive load show lower amplitude in alpha but higher amplitude in gamma.

We further calculated the spectral amplitudes separately for the four canonical frequency bands, theta (4–7 Hz), alpha (8–12 Hz), beta (13–30 Hz), and gamma (31–90 Hz), for different electrodes. The spatial patterns of the average spectral difference between task states are shown in Fig. 1b. The results exhibit some functionally and anatomically sensible features in line with previous literature: the theta effects are mainly located in the frontal region^61^, and the alpha and gamma effects are mainly located in the posterior region^62,63^. Noticeably, the effect in alpha derived from easy counting and hard counting tasks is distinctively located in the parietal region (Fig. 1b, first row, alpha), implying distinct functional engagement between them.

### Descriptive scale-free dynamics feature across task states

The spatial distributions of the average 1/*f* parameters (exponent and offset) are shown in Fig. 1c, d. Clearly structured patterns are shown in both parameters: overall, the temporal areas have lower values in both offset and exponent (thus, flatter spectrum). Clearly distinguishable patterns can be seen between high-band and low-band 1/*f* parameters, implying their differential neural substrates and the non-straightness of the 1/*f* pattern throughout the whole band. Regarding the variation of 1/*f* parameters across task states, the high-band parameters fitted from 26–90 Hz appear to be more variable across task states when compared to low-band parameters fitted from 1–25 Hz (see Fig. 1c, d, and Supplementary Fig. 1). The statistical analysis will be presented in subsequent sections.

### Variation of cross-frequency phase-amplitude coupling across task states

**Fig. 2:**
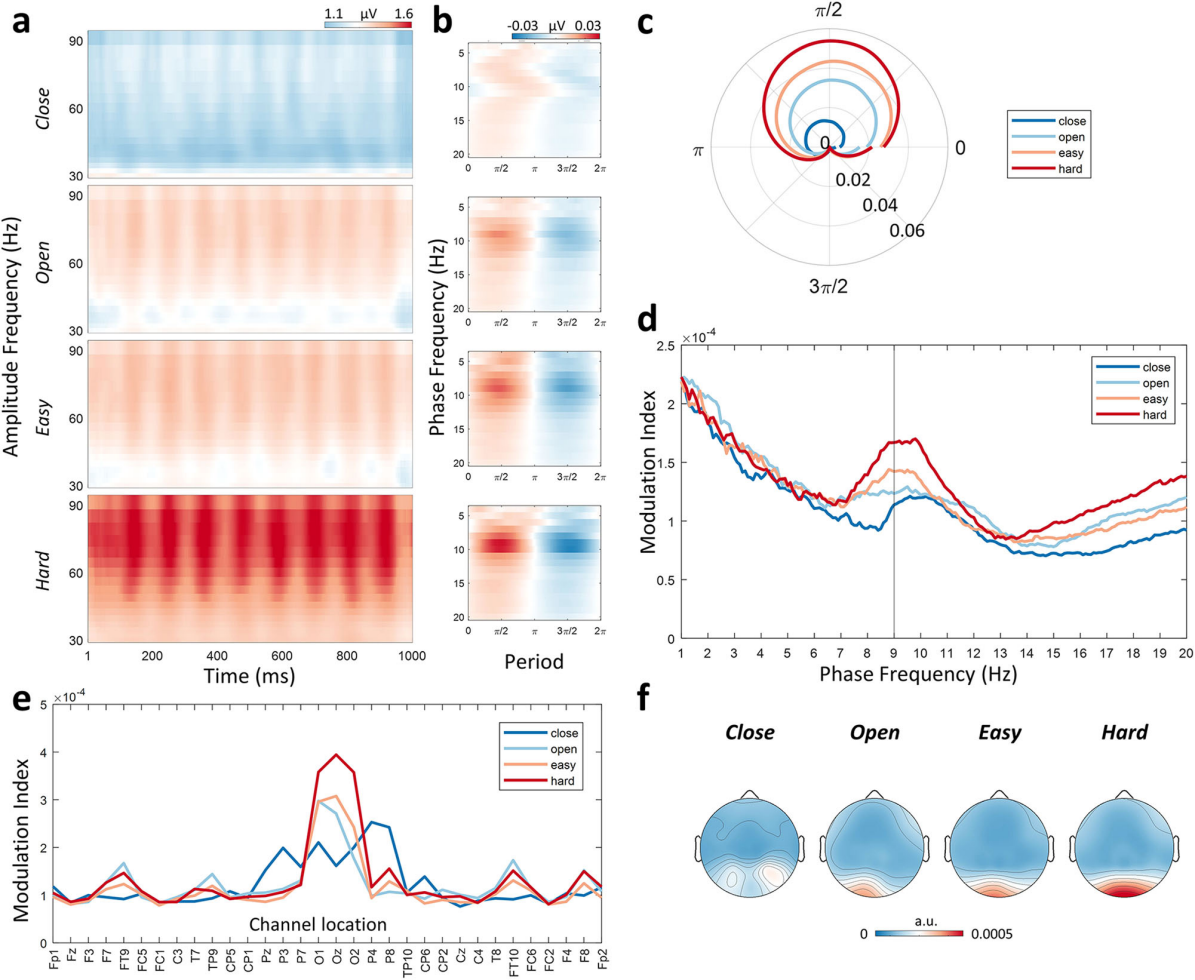
Change in phase-amplitude coupling (PAC) across task states. **a** The time-frequency representation of PAC showing that the phase of alpha (9 Hz) modulates the amplitude of gamma (31–90 Hz). This average PAC was obtained from the Oz electrode, and it appears to be modulated by cognitive load. **b** Average PAC curves calculated from Oz electrode based on the phase of low-frequency oscillation at different frequencies (4–20 Hz, with a step of 1 Hz) and the amplitude of gamma (31–90 Hz). A relatively consistent maximum strength of PAC was shown at the phase of 9 Hz oscillation. **c** The amplitude of gamma as a function of the phase of the 9 Hz oscillation represented in a polar coordinate system. This is a visualization of the result in (B) at 9 Hz. **d** The average modulation index of the PAC calculated based on the phase of different frequencies (1–20 Hz, with a step of 0.1 Hz). The amplitude for calculating PAC was always based on the gamma band (31–90 Hz). **e**, **f** The distributions of the average modulation index obtained using the phase of 9 Hz oscillation and the amplitude of gamma (31–90 Hz) from each electrode in each task state. It shows that the average modulation index was the strongest in the occipital area. Note: a.u. refers to arbitrary unit.

Next, we examined the high-order neural activity pattern “cross-frequency phase-amplitude coupling (PAC)” and how it varies across task states. The visualization of the PAC time-frequency pattern is shown in Fig. 2a–c, from which we can observe that the strength of PAC is the strongest in the hard counting task. Specifically, the amplitude of gamma band (31–90 Hz) fluctuates along with the phase of 9 Hz oscillation (strongest over electrode Oz, see Fig. 2e, f). Quantitatively, the strength of PAC estimated using the modulation index method (Fig. 2d) showed significant difference between easy counting and hard counting tasks: *t*(124) = −3.60, *p* < 0.001, resting open and hard counting task: *t*(132) = −3.35, *p* = 0.001, resting close and resting open: *t*(128) = −3.27, *p* = 0.001, but not between resting open and easy counting: *t*(126) = 0.24, *p* = 0.807. Note that the difference in the degree of freedom is due to the different numbers of outliers (see definition in Methods) excluded before conducting paired *t*-test analyses.

### Scale-free dynamics outperforms other neural features in indexing cognitive load

The results above demonstrate that the effects of cognitive load variation on neural dynamics are multifaceted, covering band-specific neural oscillations, scale-free dynamics, and cross-frequency coupling. However, an important theoretical concern is that the measurement of each neural dynamic feature is not free from the influence of others (see an in-depth elucidation of this issue from ref. ^60^). In other words, the effect revealed by one feature could be a spurious effect caused by others. For instance, variation in 1/*f* exponent causes a structural rotation effect on the spectrum^56^, which will inevitably change the result of any band-specific measurement.

**Fig. 3:**
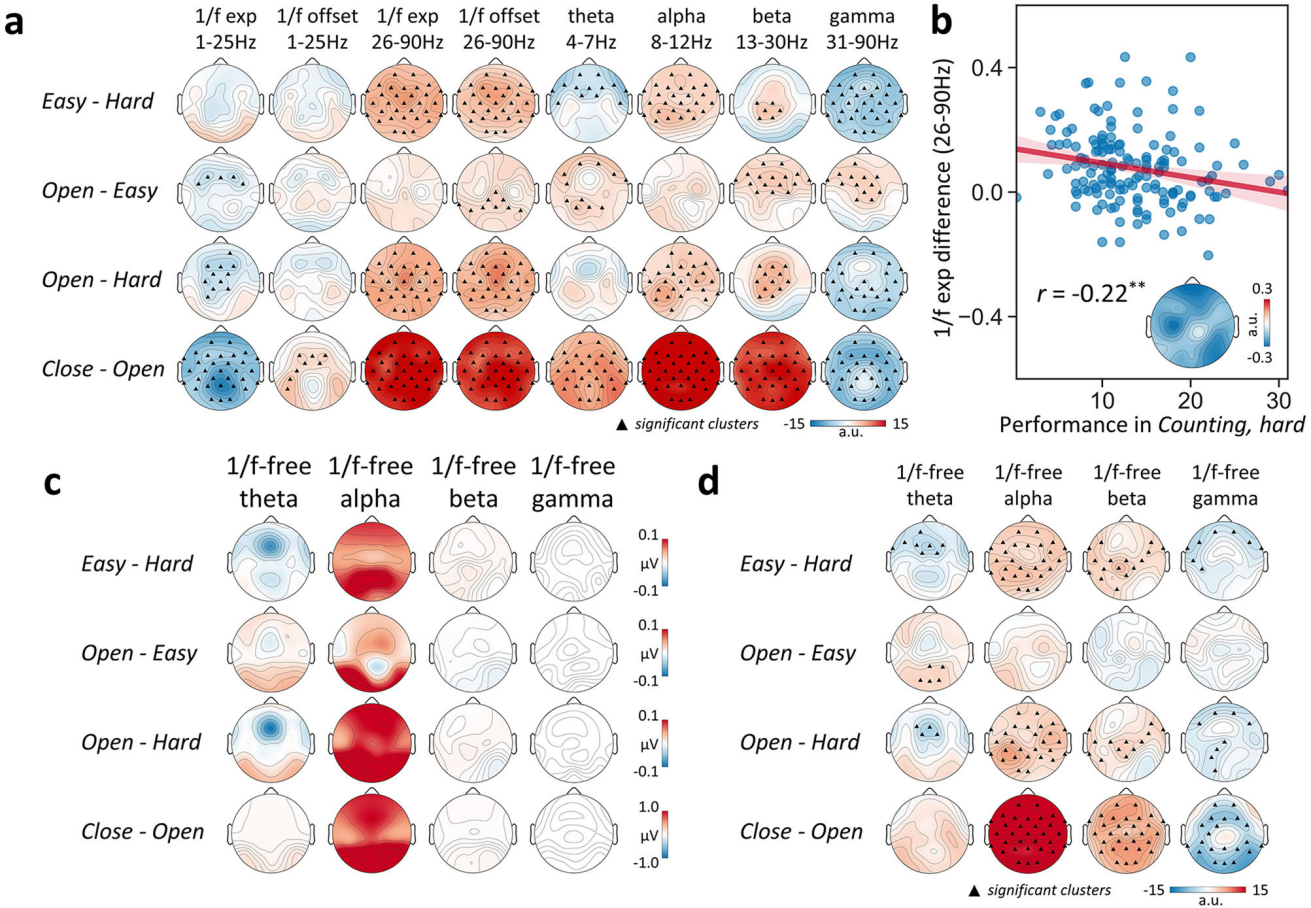
Comparison of the association strength with cognitive load across different neural dynamic features and behavioral association of 1/*f* exponent. **a** Topographies of *t* statistics indicating the statistical difference of the various neural dynamic features between different task states with clusters of markers indicating *p* < 0.05 (only significant clusters with *p* < 0.05 tested by the cluster-based non-parametric testing were shown). **b** Relationship between the behavioral performance in hard counting task and the difference in high-band 1/*f* exponent (fitted from 26–90 Hz, averaged across all electrodes) between resting open and hard counting task (*r* = −0.22, *p* = 0.006). The topography of the correlations calculated on each electrode is shown in the bottom-right corner. The 95% confidence interval of the regression line was indicated in red shade. **c**, **d** Topographies of between-task state amplitude difference (**c**) and *t* statistics (**d**) on the oscillations derived from the “1/*f*-free” spectrum. Note: a.u. refers to arbitrary unit.

To explore this issue, we proceeded to identify the dynamic feature that most strongly indicates cognitive load variation. The most essential dynamic feature underpinning an effect (here, cognitive load variation) is more likely to show the strongest statistical associations as compared to secondary dynamic features (albeit this does not rule out the functional uniqueness of the dynamic features displaying weaker associations). The strengths of the associations with cognitive load variation (represented by *t* statistics) are shown in Fig. 3a with colors denoting the magnitude and symbols denoting the significance levels. Here, the first row (easy – hard) serves to show neural effects primarily associated with cognitive load variation. For reader’s information, we also showed the differences between other pairs (2nd row: open – easy; 3rd row: open – hard; 4th row: close – open). For band-specific oscillations, most of them are significantly associated with cognitive load variation. Among the various features, the 1/*f* parameters (both exponent and offset fitted from 26–90 Hz) stand out and show the strongest association with cognitive load variation (see the first row in Fig. 3a).

The effects on the canonical oscillations shown in Fig. 3a were calculated based on the raw spectrum, which is confounded by the 1/*f* component^58,60^. To show the purer effect on oscillatory activity, we also calculated the effects on the canonical bands from the version of the spectrum that has subtracted the 1/*f* component (see Methods). The distribution of the effect size as well as the amplitude difference are shown in Fig. 3c, d. Note the slight difference between the amplitude map (Fig. 3c) and the effect size map (Fig. 3d). This is because the effect size is determined both by the amplitude difference and the data variance. The effects on the “1/*f*-free” canonical oscillations also show to be less strong than the 1/*f* parameters (Fig. 3a). In general, the “1/*f*-existent” version of effects on oscillations (Fig. 3a, especially gamma) is stronger than the “1/*f*-free” version (Fig. 3d), which confirms that the 1/*f* component has confounded the effects on oscillations obtained from the raw spectrum.

To quantitatively compare the associations with cognitive load for different dynamic features, we tabulated the effect sizes of pairwise *t*-tests from all features (see Table 1, top panel). The effect size was taken from the maximum value from all electrodes (excluding the electrodes on the very outer rim that barely covered the scalp: Fp1/2, F7/8, FT9/10, TP9/10, T7/8) to represent the greatest potential of each neural feature in indicating cognitive load variation. The modulation index (MI) was simply from the Oz electrode, as this electrode shows the strongest cross-frequency coupling (see Fig. 2e). Table 1 shows that the high-band 1/*f* exponent parameter (fitted from 26 to 90 Hz) displays the strongest effect size in the statistical association with cognitive load variations (see particularly the difference between easy and hard, i.e., first row; other pairs of task state are shown for reference). This means that this parameter is the strongest neural indicator in indexing cognitive load variation amongst all neural features examined in this study. To validate that the participants with incorrect answers in the hard counting tasks actually performed the task rather than giving arbitrary answers, we also presented the results above separately for participants reporting correct and incorrect answers in the hard counting task (see Table 1, middle and bottom panels). The results showed high consistency across the two subsets of participants, especially for the high-band 1/*f* parameters (Table 1). Furthermore, we did the same split analysis for males and females, which again showed consistency across genders (see Supplementary Tables 1, 2).

**Table 1:** Maximum effect sizes (Cohen’s d) of paired *t*-tests between task states for different dynamic features.

	1/*f* parameters 1–25 Hz		1/*f* parameters 26–90 Hz		theta 4–7 Hz	alpha 8–12 Hz	beta 13–30 Hz	gamma 31–90 Hz	MI
	exp	offset	exp	offset					
All participants (*N* = 152)									
Easy vs. Hard	0.26	0.19	**0.65**	**0.62**	−0.35 −0.41	0.47 0.46	0.42 0.45	−**0.57** *−*0.25	−0.25
Open vs. Easy	−0.35	−0.20	0.21	0.30	0.37 0.28	0.27 0.28	0.39 −0.20	0.34 −0.15	−0.15
Open vs. Hard	−0.44	−0.26	**0.74**	**0.77**	−0.35 −0.49	**0.58** **0.65**	**0.60** 0.33	−**0.58** −0.26	−0.36
Close vs. Open	−**1.12**	0.43	**1.35**	**1.30**	**0.84** 0.39	**1.50** **1.49**	**1.25** **0.69**	−**0.83** **−0.80**	−0.12
Participants reporting correct answers in the hard counting task (*N* = 96)									
Easy vs. Hard	−0.29	0.21	**0.63**	**0.63**	−0.36 −0.39	0.43 0.41	0.43 0.41	−**0.60** −0.26	−0.32
Open vs. Easy	−0.44	−0.32	0.28	0.30	0.36 0.37	0.27 −0.25	0.39 −0.25	0.36 −0.22	−0.02
Open vs. Hard	−**0.55**	−0.42	**0.70**	**0.76**	−0.39 −0.40	**0.54** 0.32	**0.58** 0.32	−**0.50** −0.27	−0.35
Close vs. Open	−**0.96**	0.47	**1.47**	**1.39**	**0.86** 0.43	**1.40** **0.68**	**1.15** **0.68**	−**0.86** −**1.00**	−0.27
Participants reporting incorrect answers in the hard counting task (*N* = 56)									
Easy vs. Hard	0.34	0.23	**0.80**	**0.74**	−0.35 −**0.53**	**0.50** **0.55**	0.40 0.49	−**0.81** −0.43	−0.34
Open vs. Easy	−0.36	0.32	0.32	0.39	0.37 −0.25	0.31 −0.34	0.41 −0.37	0.42 0.18	0.10
Open vs. Hard	−0.44	0.31	**0.81**	**0.83**	0.30 −**0.63**	**0.65** **0.69**	**0.66** 0.41	−**0.68** −0.46	−0.21
Close vs. Open	−**1.49**	0.47	**1.37**	**1.31**	**0.86** 0.32	**1.71** **1.71**	**1.49** **0.88**	−**0.85** −**0.72**	−0.26

Here, d = t/$\sqrt{n}$ where *n* is the number of participants. The two values for the canonical bands correspond to the result obtained from the raw spectrum (top) and the 1/*f*-removed spectrum (bottom). MI is based on the phase of 9 Hz.

Bold values denote effect sizes larger than 0.5 (medium effect size); exp refers to exponent; MI refers to Modulation Index.

We next conducted an LMM analysis (see Methods) to examine how the various neural features uniquely predict cognitive load variation between easy and hard counting tasks. Here, the neural features that display strong collinearity were excluded (see Methods), and all the independent variables (IV) have been transformed to achieve normality. The final variance inflation factor and transformation used for each IV are shown in the table. In line with the result in Table 1, the LMM results shown in Table 2 confirm the unique effects of high-band 1/*f* exponent, theta, and alpha, i.e., they are not epiphenomena of each other.

**Table 2:** Fixed effects estimated using linear mixed model (LMM).

	*b*	SE	CI (95%)		*t*	VIF	Method used for normality transformation
			Low	High			
(Intercept)	3.70	0.29	3.14	4.26	12.97		-
gender	0.00	0.06	−0.13	0.13	−0.03	1.11	-
**high-band 1/*f*** **exponent**	−0.54	0.21	−0.95	−0.12	−2.54*	1.39	-
**theta**	0.54	0.19	0.17	0.90	2.88**	2.13	-
**alpha**	−0.28	0.09	−0.46	−0.10	−3.02**	2.38	log(x)
**gamma**	0.35	0.15	0.05	0.65	2.30*	1.92	log(x)
MI	0.00	0.00	0.00	0.00	0.31	1.25	(log(x))^2^

*b* Co-efficient in the linear mixed model, *SE* Standard error, *CI* Confidence interval, *VIF* Variance inflation factor, *t* t statistics, ***p < 0.001; **p < 0.01; *p < 0.05. Bold values denote statistical significance at the *p* < 0.05 level.

### Prediction of cognitive load by neural dynamic features based on machine learning

**Fig. 4:**
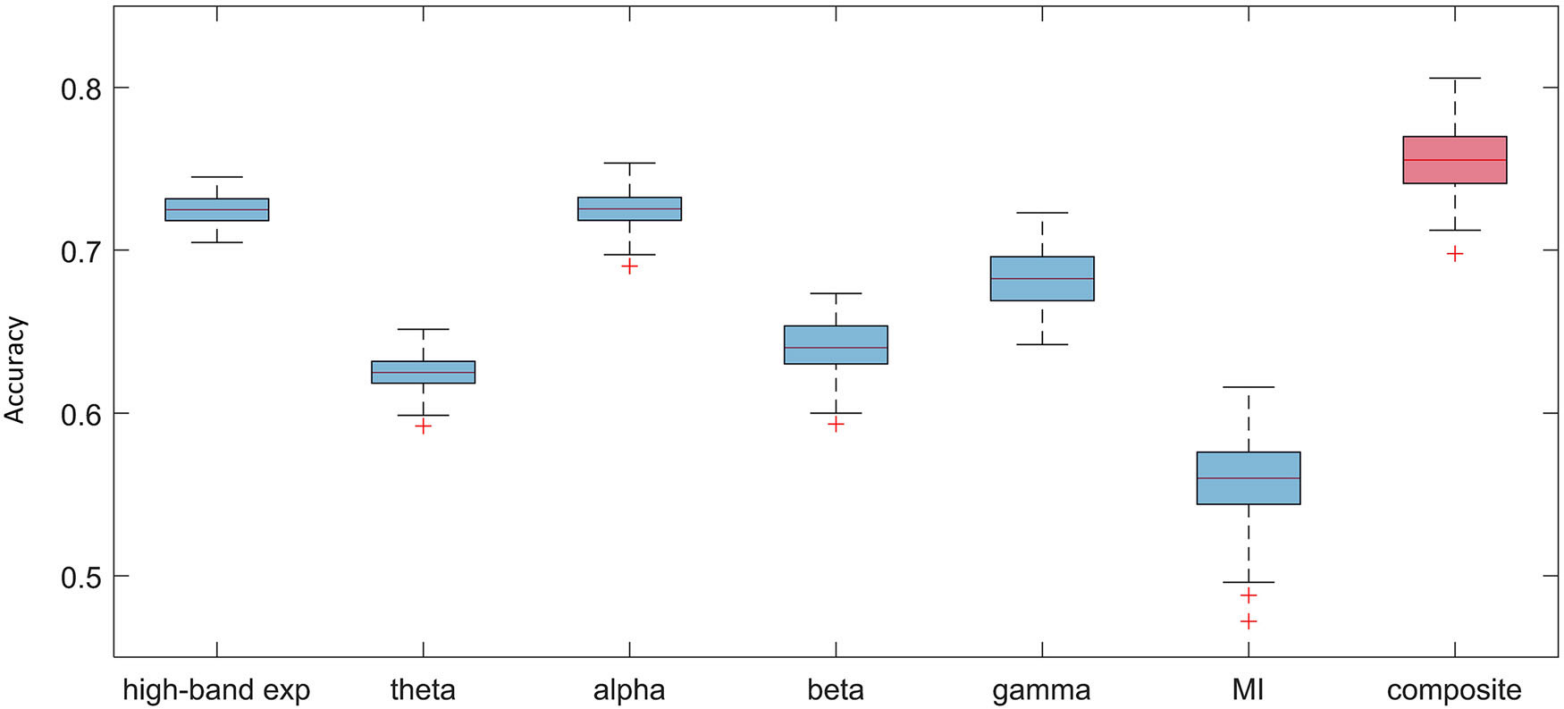
Prediction accuracies based on different neural dynamic features. The boxplot of the prediction accuracies from 100 permutations. The line splitting the box represents the median. The top edge of the box represents the upper quartile value and the bottom edge represents the lower quartile value. The upper and lower whiskers represent the maximum and minimum values of the data, respectively. The red crosses outside the boundary of whiskers represent the outliers. The composite indicator (last column) combines high-band 1/*f* exponent, alpha, theta, and beta. Note: exp refers to exponent, MI refers to modulation index.

We used a support vector machine (SVM)-based classifier to demonstrate that different features contain complementary information about cognitive load (see Methods for the machine learning details). The basic rationale is that if different features contain non-redundant information, a combination of them will achieve a higher prediction accuracy than any single feature. As described in Methods, the feature data used were the difference between the second and the first counting task, and the variable to be predicted was the temporal order of two (easy and hard) counting tasks. This approach was used to avoid the overwhelming variance of baseline neural features across participants. Figure 4 shows the results of prediction accuracy (based on test data) for the key individual neural dynamic features (1st to 6th column) and for the composite feature that shows the highest performance (last column).

### Scale-free dynamics predicts behavioral performance

Given the close associations between the neural dynamics features and cognitive load, it is possible that these neural dynamic features are able to predict individuals’ behavioral performance^64^. To explore this relationship, we calculated the correlation between the behavioral performance in the hard counting task and the variation of neural dynamic features between task states with different cognitive loads. For the neural dynamic feature, we examined the strongest neural indicator of cognitive load shown in Fig. 3 and Table 1: high-band 1/*f* exponent. We found that the difference in high-band 1/*f* exponent (electrodes-averaged) between resting open and hard counting task states significantly predicts the behavioral performance in the hard counting task (*r* = −0.22, *p* < 0.01; see Fig. 3b; results based on 1–30 Hz and 30–90 Hz can be found in Supplementary Fig. 5b): individuals with better counting performance have their high-band 1/*f* exponent less altered by the hard counting task. Furthermore, the correlation values appear to be consistently high across the whole scalp (see Fig. 3b and Supplementary Fig. 5b). The association is robust across gender (Supplementary Fig. 2a, b). However, the association exists only when the difference in high-band 1/*f* exponent between resting open and hard counting was used, but not when the difference between easy counting and hard counting was used (See Supplementary Fig. 2c, d).

## Discussion

The present study comparatively analyzed multiple facets of neural dynamics associated with variation of tasks aimed to simply change the internal workload of cognitive activities (termed endogenous cognitive load here). It was found that variation in endogenous cognitive load alters the brain’s dynamics across a wide range of characteristics covering the aspects of band-specific oscillations, scale-free dynamics, and cross-frequency phase-amplitude coupling. Amongst them, the scale-free dynamics appears to be the predominant effect target of cognitive load variation. And the scale-free dynamics is shown to be indicative of individual differences in behavioral performance in mental calculation.

The relationship between neural signals and cognitive load has been a well-attended topic in applied cognitive neuroscience fields, such as educational neuroscience and neuroergonomics. Earlier explorations in this line have focused on specific and simplistic neural indicators such as band-specific oscillation power or amplitude of event-related potentials^8,11^. These explorations follow a traditional assumption that there is a one-to-one mapping between cognitive activities and neural dynamic features; that is, a specific cognitive activity engenders a specialized neural dynamic activity that plays as a neural representation of it^65^. A large body of research has, explicitly or implicitly, adopted this view as the foundation for studying specific neurocognitive questions, for instance, identification of the specific cognitive signature of band-specific oscillations or event-related brain response components^37,61,66^.

However, from the neural system perspective, it has been firmly established that the brain is a highly complex, self-sustaining, and active dynamical system with constantly interacting sub-functional modules that simultaneously maintain a repertoire of cognitive functions^67^. Due to the cohesiveness of the complex dynamical system, an external event driving a change of cognitive states may lead to widespread alternation of the internal spontaneous dynamic state rather than separately affecting a clearly segregated sub-process or engendering an additional activity that is independent of the ongoing activity^68^. In line with this notion, our current work demonstrates that cognitive load variation alters multiple neural dynamic features, including narrow-band and broad-band oscillation power, aperiodic neural dynamics, and cross-frequency coupling. The major implication is that application of neural dynamics-based cognitive load monitoring should consider an integrative algorithm that involves and exploits multifaceted neural dynamic features.

Upon the confirmation of the multifaceted effects of cognitive load variation on neural dynamics, we now discuss the potential functional correlates of those dynamic changes incorporating the existing theories and findings in the literature. The subjective perception of certain mental state changes commonly described as magnitude variation in a single dimension (e.g., cognitive load, pain) is most likely not resulted from a single cognitive process. Instead, it is more likely to be composed of an amalgam of multiple processes (both task-general and task-specific) associated with multiple neural dynamic characteristics. First, the effect on theta band was exclusively located in the frontal region (Figs. 1b,  3a). This is fully in line with the established feature of the frontal theta that reflects top-down cognitive control^61^. The continuous mental calculation task requires prolonged concentration on the task–in other words, constant active cognitive control. This may explain the presence of stronger frontal theta in the hard counting task.

Second, the effect on alpha is shown to be in posterior areas. The alpha oscillation is the most prominent neural dynamic indicator that indexes universal task engagement in a way that task demand is correlated with alpha power reduction^37,69–72^. That said, cognitive load variation will certainly be associated with the alpha variation. As a fundamental component of neural functional dynamics, alpha is likely a thread that is functionally separate from (but could interact with) other neural dynamic effects.

We will skip the discussion on the beta band effect as the effect (although significant) did not show a highly structured pattern, and it is hard to link the existing literature about the relationship between beta and cognitive load. Moreover, the beta band appeared to be the axial point of the rotation of the 1/*f* component and also the point that separates the 1/*f* line into two discontinuous segments.

Third, the effect in gamma is strong and widespread (Fig. 3a). Functionally, this may be linked to the process of increased intensity of neuronal firing activity that contributes to the high-frequency band^73–76^, which is a neural representation of computations engaged in generic task processing. It is thus another form of indicator of task engagement with different functional roles from alpha and theta bands. To sum up, the potential neural and cognitive processes involved in the load variation may include cognitive control (theta), general maintenance process (alpha), and increased neuronal firing (gamma).

Finally, there is the scale-free dynamics of which the functional signature needs to be interpreted. It is worth reiterating that the scale-free dynamics is found to be the dominant neural indicator (Table 1) of the cognitive load variation in the present study. Below we attempt to incorporate the existing theories about neural scale-free dynamics for the functional interpretation. In complex system theories, scale-free dynamics has been commonly proposed to be a functionally optimal state in multiple domains^77^. Prior to a task, the neural system is ‘resting’ in a functionally optimal state preparing for the task. During the task, the scale-free state is disrupted, and the dynamics is absorbed into a state that is functionally specific or task-related^4,57^. This mechanism is at a fundamentally different level from other specific dynamic features as listed above and appears to be domain-general (similar to the alpha band effect). The surprising result here is that this fundamental effect turned out to be the strongest change amongst the neural dynamic features associated with cognitive load variation.

When examining the dynamic effects in multiple aspects, it is pivotal to investigate the interdependence of different aspects (features). An obvious reason is that an observed effect can be an epiphenomenon of another more essential effect. In the present work, a concrete example is that the rotation of the 1/*f* spectral pattern, which is a key feature of the scale-free dynamics, will result in variation of band-specific oscillation power measured across the whole band (except for the fulcrum point)^56^. Our main hypothesis about the multifaceted effects of cognitive load variation on neural dynamics will be critically challenged if the observed multifaceted features originate from the same underlying factor.

Regarding this point, we provided the following arguments that support the independence of the dynamic effects examined. First, the spatial features of different effects are distinct from each other. The difference between easy and hard counting should be of primary interest here. The effect on the theta band shows a very clear frontal distribution (Figs. 1b, 3a), which is consistent with the classic frontal theta component^28,78^ and is not shown in other dynamic features. For alpha, the effect shows a posterior (in the amplitude, Fig. 1b) and a parietal (in the effect size, Fig. 3a) distribution, making it substantially different from the theta effect. The effects on the gamma band, on the contrary, appear to be more similar to the effects on the scale-free dynamics. This is likely due to the broadband feature of gamma, which is closely entangled with the scale-free component. Their strong entanglement is also reflected by the result that the removal of 1/*f* component from the raw spectrum considerably changes the effect size of gamma but less others (Fig. 3a, d). It remains an open issue here as to how to properly differentiate the broad gamma band component and the scale-free component. Second, the LMM results (Table 2) also showed that multiple features contribute unique variance to cognitive load variation. Finally, the machine learning approach clearly showed that the integration of multifaceted neural dynamic features reaches a higher performance in predicting cognitive load variation, which is one of the key points aimed to be conveyed in the present work. In sum, the data support that the cognitive load variation engenders wide-ranging effects on multiple facets of neural dynamics that are not likely to be epiphenomena of a single common origin. Further support from neuroanatomical studies can help to solidify this claim.

Cross-frequency phase-amplitude coupling (PAC) is a unique and functionally relevant neural phenomenon in the brain^79–81^. This is a high-order dynamic feature that concerns the relation between different dynamic activities or processes. In line with previous findings^54,81^, we also found that cognitive load is associated with PAC. Specifically, the PAC is the strongest in the task state of hard counting, i.e., the phase of the low frequency of 9 Hz increasingly modulates the amplitude of high frequency (31–90 Hz) when the cognitive system is more intensely loaded. According to the proposed theory of the functional roles of PAC^52^, this result may be interpreted as an enhanced coordination between different neural processes during high-load states. This adds an additional layer to the pool of dynamics effect imposed by cognitive load variation, further revealing that the effect of mental state changes on neural dynamics traverses multiple orders and dimensions of the dynamic brain activity.

Here, we proposed that the cross-frequency coupling could be an interface between scale-specific neural dynamics (oscillation) and scale-free dynamics, thus forming a candidate mechanism that integrates simple and complex dynamics see also^55,82^. The major rationale is that the gamma band activity is a broadband activity and has substantial overlap with the scale-free dynamics^76^. It has been previously proposed that, technically, the gamma band neural effect could be a spurious effect of broadband activity^83^. Besides, the commonly referred 1/*f* feature in the spectrum actually only applies to the frequency segments above a low cutoff, i.e., in the medium-to-high frequency band^82^. Therefore, it is possible that the low-frequency oscillation actually modulates the scale-free dynamics, not high-frequency oscillations like gamma. Mechanistically, the coexistence and interaction of oscillations and scale-free dynamics have been demonstrated in computational models^84,85^, which provides the foundation for the biological plausibility of our proposal. However, the current result is not able to answer the functional signature of the potential coupling between neural oscillations and scale-free dynamics, which is an important question to be addressed in the future.

Despite numerous studies on the cognitive association of scale-free dynamics, there remains an open issue in the very definition of scale-freeness. The concept of “scale-free” originates from simplistic physical models^86,87^ and serves as a landmark for critical dynamics. Although it has since been widely proposed that the brain functions optimally at critical dynamic states^4^, a strictly-defined power-law pattern does not exist in brain activity patterns^58,88–90^. Instead, the power-law pattern only exists within confined frequency ranges, and different ranges may contain power-law patterns with different exponents^55,58,89,91^. Therefore, the estimation of 1/*f* spectral pattern is usually selected ad hoc, which differs across studies, e.g., 1–40 Hz^60,92^, 1–20 Hz^92^, 2–24 Hz^93^, 4–30 Hz^69^, 1–30 Hz^94^, 20–40 Hz^92^, 30–45 Hz^89^, and 1–50 Hz^95^. This also highlights a fundamental issue in the use of the term ‘scale-free’ throughout the article. The terminology of ‘scale-free’ actually goes against the idea of estimating relevant parameters in a band-confined (thus, scale-not-free) manner. This issue exists in all similar studies in the literature. One argument for this issue is that researchers can use reality-restricted data features to estimate a theoretical construct. A specific frequency band needs to be specified for estimating the theoretical construct of scale-freeness owing to the fact that the low end and high end of the spectrum are shaped by biological or technical constraints^88^.

In this study, we fitted the 1/*f* pattern separately over two frequency ranges split at 25 Hz. As mentioned in Methods, this choice was made after observing the discontinuity of the 1/*f* profile in our data at somewhere within the beta range between 20 and 30 Hz (see Fig. 1a). The point of 25 Hz was also approximately at the rightmost bound of the beta hump in the spectrum (Fig. 1a), so FOOOF does not need to split a hump which may cause more technical error^88^. We have conducted additional analyses to show that the main results of this study are not sensitively dependent on the specific cutoff value: replacing it with 30 Hz did not change the major pattern and conclusion (see Supplementary Figs. 3–5 and Supplementary Table 3).

Further to the discontinuity issue in the 1/*f* pattern, we expected that the 1/*f* components in the two segments (1–25 Hz and 26–90 Hz) should possess certain fundamental differences at the functional level. First, the scalp distributions of their associations with cognitive load variation are quite different (Figs. 1c, d, 3a), indicating different anatomical bases. Second, high-band 1/*f* parameters are more associated with cognitive load. The direction of the association, i.e., lower exponent associated with higher load, is consistent with previous studies^55–59,69^. This band-related difference is also in line with one of our recent studies with a large sample (*n* = 102), demonstrating that high-band 1/*f* parameters exhibited a stronger association with controlled cognitive processing^96^. These results solidly reveal that the 1/*f* dynamic feature from the two different bands is clearly not a unitary dynamic feature, echoing previous claims that scale-free dynamics in different frequency ranges might result from different generative mechanisms^76,82,89^. It has to be noted that such dependence of scale-free dynamics on frequency band is in conflict with the intrinsic nature of scale-freeness. This issue remains to be addressed in the future.

The discontinuity issue may be due to a mixture of different mechanisms that may generate the 1/*f* feature, such as low-pass filtering of tissues^97,98^, brain network properties^90^, and emergent critical dynamic properties^99,100^. In addition, the tonic neuronal spiking activity could also significantly contribute to the broadband high-frequency spectrum with a 1/*f* pattern^101,102^. This heterogeneity issue poses an additional requirement in data analysis: it is not just that the scale-free component has to be isolated from the band-specific oscillations^58,60,103,104^, but also that the scale-free component itself may be composed of multiple dynamic processes that need to be disentangled. Our results firmly showed the functional and anatomical dichotomy of the scale-free dynamics between low and high bands.

Several limitations remain in the current study. The first one is that the high-frequency data (e.g., gamma band) could be contaminated by muscle and eye-movement-related artifacts^105–109^. As such, neural effects found from the gamma band could be generated by non-neural factors, such as different saccade frequencies^106^. To address this issue, more advanced methods such as co-registration of EEG, eye-tracking, or EMG would need to be adopted in order to thoroughly remove the artifacts. While the current study is not able to fully address this limitation, the data did support that the gamma-related effect is not totally originated from artifacts: the PAC between gamma and low-alpha band clearly exists, indicating that the gamma power is clearly modulated by low-frequency oscillations. There is no theoretical foundation for the link of such PAC to muscle artifacts.

Second, there remain some inherent limitations in the task design. In the context of applied research, the cognitive load has been commonly treated as a homogeneous mental variable associated with a scalar magnitude. However, from a basic cognitive process point of view, it can be a highly composite variable that contains many sub-processes. In our design, we assumed that the three tasks, eye-open resting, easy counting, and hard counting, exert monotonically increasing cognitive load. However, this cognitive load refers to the intensity of neurocognitive activity in a generic sense, not to a specific cognitive process that determines the load (e.g., more information chunks in working memory). The composition of cognitive activity in the three tasks can be highly heterogeneous. In the eye-open resting state, the cognitive process can be anything due to free mind wandering. Still, we believe the resting state was not a high-load state because the participants were instructed to relax. For the two counting tasks, although it is reasonable to assume that the intensity of cognitive activity is higher in the hard counting mode, the specific cognitive process is not a homogeneous one that simply varies its intensity: in the easy counting mode, the participant may just go through a rhythmic automatic action without any arithmetic process; in the hard counting mode, arithmetic processes (complicated per se) are involved. To sum up, it would be more desirable to manipulate a single cognitive process in its intensity to achieve different levels of cognitive load while making other processes under control to make the results more interpretable in a cognitive sense. That said, the current result of the multifaceted neural effect reflects a variation of cognitive load in a generic sense of activity intensity, not a specific identifiable cognitive process.

Third, the current design did not introduce a component to monitor or assess the genuineness of the task performance, particularly in the hard counting task. While we can assume that the erroneous answer from the hard counting task was mainly due to task difficulty, it is equally probable that the participant did not genuinely perform the task. Related to this issue, we have separately analyzed the neural effects and showed consistency in them across participant groups with correct and incorrect answers. Specifically, effect sizes of the nine neural indicators calculated for the same condition were comparable between the two participant groups; more importantly, the high-band 1/*f* parameters turned out to be the most robust and effective neural indicator of cognitive load variation between different task states for both the two participant groups (Table 1). This validation analysis demonstrated that the two groups went through similar cognitive activities. However, in future studies, the task should be introduced with mechanisms to ensure performance genuineness.

In conclusion, the present work presents a detailed demonstration that different degrees of cognitive load are associated with wide-ranging effects on neural dynamics in many facets with a key role in scale-free dynamics. The complexity in the relationship between neural dynamics and cognitive dynamics reveals the intricate linkage between the neural dynamic system and the mental system. In other words, it also clearly reveals the issue in attempting to identify a simplistic neural indicator to indicate complex cognitive variables such as cognitive load. One implication is that the study of cognitive effects on neural activities should adopt a more holistic view of the neural dynamics incorporating multifaceted (rather than single) dynamic features embedded in the spontaneous neural activity.

## Methods

### Participants

A total of 156 healthy young adults in Hong Kong completed the experimental tasks in this study. Four participants were excluded due to non-cooperative behaviors or technical problems during the experiment. All included participants (42 men and 110 women; age 24.20 ± 3.93 years) reported normal or corrected-to-normal vision and no history of mental disease and gave informed consent. Note that the unbalanced gender may create gender bias, so we also present the major results separately for different genders to show consistency. The study was approved by the Human Research Ethics Committee (HREC) of the University of Hong Kong. The experiment was conducted in a sound-attenuated room in which the participants performed a series of cognitive tasks during EEG recording. The EEG signals were recorded by a 32-channel BrainAmp DC amplifier (Brain Products, Germany) referenced to the GND electrode located at the mid-point between Fp1 and Fp2 at a sampling rate of 1000 Hz and were stored using BrainVision PyCorder. The electrodes were mounted on an elastic cap (Easycap, Brain Products, Germany) in accordance with the 10–20 system^110^.

### Task

Three experimental tasks were used in the present study, and the order of administering these three tasks was counterbalanced across participants. The first task was a resting state task during which the participants were instructed to stay relaxed with eyes open for 60 s (natural eye blink allowed) and then closed for another 60 s. The second and third tasks were two backward counting tasks in easy and hard modes, respectively. In the easy counting task, the participants were instructed to silently (without mouth movements) count down from 100 by deducting 1 each time for 60 s. In the hard counting task, the count-down was from 300 and the deduction number was 7, and the duration was also 60 s. Like the eyes-open resting state task, the two backward counting tasks were also stimulus-free and performed with open eyes. After completing each backward counting task, the participants were asked to type the final number they arrived at into the computer. The two counting tasks used in this study were adapted from a classic task paradigm of serial subtraction that has been widely used for mental status examination^111,112^ and for imposing different levels of cognitive load for cognitive research^112,113^.

The three tasks generated four mental states, three of which were assumed to represent different levels of endogenous cognitive load. Specifically, the eyes-open resting state is supposed to impose a low cognitive load because no explicit task processing was involved. The two counting tasks were assumed to create two different levels of cognitive load. For the eyes-closed state, we did not intend to use this state to represent any variation of the cognitive load because the action of eye-closing drastically changes the neural dynamics. Instead, this state mainly serves for sanity-checking the EEG data in the present study ("Berger effect"^114^). We will still present the difference between the eyes-closed state and others for readers’ information, but not for addressing the main research questions. In sum, the tasks were assumed to generate three different levels of cognitive load without interferences with major sensorimotor processes: (1) eyes-open resting state (minimal cognitive load), (2) easy counting (low cognitive load), and (3) hard counting (high cognitive load).

### EEG data preprocessing

EEG signals were preprocessed and analyzed using MATLAB and EEGLAB plugin^115^. Each participant’s EEG data encompassed by each of the three tasks were cut out and down-sampled to 250 Hz, high-pass filtered above 1 Hz (zero-phase, non-causal, filter order: 827 data points, corresponding to 3.3 s, cutoff frequency at −6 dB: 0.5 Hz). High-pass filtering at 1 Hz or above is a common pre-processing practice and has been demonstrated to be a prerequisite for good ICA decomposition^116–118^ (also see EEGLAB tutorial). Electrodes identified as outliers (with variance larger than 4 median absolute deviations (MAD) across all electrodes) were interpolated in EEGLAB. Afterward, the data were referenced to the common average offline. The processed data for the three tasks were then concatenated and decomposed using Independence Component Analysis (ICA). Next, the artifacts were identified and removed automatically using MARA with a default cut-off probability of 50%^119^.

### Behavioral performance

The behavioral performance in the two backward counting tasks was evaluated as the total number of subtractions performed within the task duration (i.e., 60 s). For instance, if a participant arrives at 31 in the easy counting task, his/her performance in this task will be (100-31) = 69; if a participant arrives at 237 in the hard counting task, his/her performance in this task will be (300-237)/7 = 9. It is possible to have non-integer numbers in the performance of the hard counting task because of errors made in between. The counting frequency was calculated as the total number of subtractions divided by 60 (total seconds), which represents how many times the participant counted in every single second on average.

### Amplitude spectrum across frequencies

The amplitude spectrum was calculated using discrete Fast Fourier Transform (*fft* in MATLAB) using Bartlett’s method on each electrode, task, and participant. The 60-second EEG segment was divided into 60 1-second epochs, and the amplitude spectrum from each epoch was calculated and the average spectrum (1–100 Hz) across the 60 epochs was obtained. The AC artifact at 50 Hz was notched out by replacing the amplitude at 50 Hz with the average of amplitudes at 49 Hz and 51 Hz.

Based on the amplitude spectra obtained above, the amplitudes of the four canonical oscillations, i.e., theta (4–8 Hz), alpha (9–12 Hz), beta (13–30 Hz), and gamma (31–90 Hz), were calculated using the average amplitude across their corresponding frequency ranges. We did not include delta because the cut-off frequency filtering at 1 Hz significantly diminished the power of the low-frequency end and Bartlett’s method based on relatively short time windows also lost significant low-frequency information. The oscillation amplitude calculated in this way — although commonly used in the field — does not take into consideration the contribution of the 1/*f* component underneath the assumed oscillations and thus has been increasingly considered problematic^58,60,88^. We conducted this conventional calculation in order to make our results comparable to the previous studies; we also calculated a 1/*f*-free version of canonical oscillation amplitudes later (see below).

### Parameterization of 1/*f* dynamics

The parameterization of the 1/*f* spectral pattern was conducted using the FOOOF method^58^ to fit the spectral curve with a linear component (in the log-log scale) representing the 1/*f* trend and several Gaussian humps representing the band-specific oscillations. FOOOF was separately applied on the low frequency (1–25 Hz) and high-frequency range (26–90 Hz). Accordingly, two sets of 1/*f* parameters (exponent and offset) were obtained. This is a heuristic decision based on the following considerations: (1) there is a visually discernable discontinuity of the linear trend in our average spectra (in log-log scale) at around 25 Hz (see Fig. 1a); (2) it is not recommended to fit 1/*f* component on a frequency range that splits an oscillation hump^88^, and the beta hump in our data ended at around 25 Hz (see Fig. 1a). For convenience, the 1/*f* parameters estimated from 1 to 25 Hz are referred to as low-band 1/*f* parameters, and the ones estimated from 26 to 90 Hz are referred to as high-band 1/*f* parameters throughout the article. For the fitting, the maximum number of oscillation peaks allowed was specified as 2 and 0 for the low and high bands, respectively. The reason is that normally no oscillation hump exists in the high band, and the humps in the low band were commonly seen in alpha and beta bands. The method of split fitting has also been performed in previous research to obtain a better local fit^92^.

Considering the short duration (60 s) of EEG data for each task state, we also estimated the reliability of the 1/*f* parameters. In doing so, we obtained the 1/*f* parameters from the first and second halves of the data (30 s each) on each electrode and calculated the reliability as the cross-session *Pearson* correlation^120^, which shows to be high (see Supplementary Table 4). The goodness-of-fit of the 1/*f* fit estimated by R^2^ (percentage of variance explained by the model) was also high: 0.94 (resting, close), 0.96 (resting, open), 0.95 (counting, easy) and 0.95 (counting, hard) for the low band and 0.86 (resting, close), 0.80 (resting, open), 0.80 (counting, easy), 0.75 (counting, hard) for the high band (averaged across electrodes and participants).

After fitting the spectra with the FOOOF method, the 1/*f* components fitted from the two bands were subtracted from the original spectra, and the resultant spectra were used to calculate the amplitude of canonical oscillations as described above, thus generating a 1/*f*-free version of the oscillation amplitudes. It has to be noted that this 1/*f*-free version may have negative values of oscillation amplitude in individual participants due to the nature of the fitting methodology, but it can still serve as a valid indicator of individual differences.

### Phase-amplitude coupling

The phase-amplitude coupling (PAC) between low- and high-frequency bands in this study was characterized in both qualitative and quantitative ways described as follows.

The first way of characterization focused on qualitative visualization rather than quantification of the PAC. First, the time-frequency representation (TFR) was calculated from each 1-second epoch by applying wavelet transformation based on Morse wavelet with the symmetry parameter (gamma) equal to 3 and the time-bandwidth product equal to 60. The moduli of the complex values from wavelet transformation were obtained as the TFR. The 60 TFRs from the 60 1 s segments were then averaged after synchronizing to their phases at a specific low frequency (one from 4 Hz to 20 Hz) as follows: the TFR from epochs with earlier phases will be moved rightward, and the TFR from epochs with later phases will be moved leftward, according to their phase values. In this way, the modulation of high-frequency amplitude by the low-frequency phase can be revealed after averaging the synchronized TFRs^81^. This analysis was done on each electrode and low frequency (from 4 Hz to 20 Hz) separately. We further characterized the relationship between the amplitude of gamma (31–90 Hz) and the phase of the low frequency by folding the average TFR with f folds (f is the value of the low frequency in hertz) and averaging the folded TFR. The final average TFR was averaged over the gamma band (31–90 Hz) to obtain the gamma amplitude-low frequency phase relationships. The analysis was done at different low frequencies in order to examine the systematic dependence of PAC on the low frequencies.

The second way of characterizing PAC was based on quantification. In doing so, the strength of PAC was calculated using the modulation index^121,122^ based on the following procedures. (1) The preprocessed EEG signals were first filtered at two bands: the low-frequency band from which the phase is to be obtained, and the high-frequency band from which the amplitude is to be obtained. (2) Hilbert transform was applied to the two filtered signals to obtain the low-frequency phase and the high-frequency amplitude, respectively; (3) the obtained high-frequency amplitudes were discretized into 18 bins according to the low-frequency phases. (4) the MI was then calculated as the divergence of amplitude distribution (over the 18 bins) from uniform distribution based on the formula below.

1 $${MI}=\frac{{D}_{{KL}}\left(P,U\right)}{{{\log }}\left(N\right)}=\frac{{{\log }}\left(N\right)-H\left(P\right)}{{{\log }}\left(N\right)}=\frac{{{\log }}\left(N\right)-{\sum }_{j=1}^{N}P\left(j\right){{\log }}\left[P\left(j\right)\right]}{{{\log }}\left(N\right)}$$

2 $$P\left(j\right)=\frac{{ < {A}_{{f}_{A}} > }_{{\varphi }_{{f}_{p}(j)}}}{{\sum }_{k=1}^{N}{ < {A}_{{f}_{A}} > }_{{\varphi }_{{f}_{p}(k)}}}$$

where *N* is the number of phase bins; ${D}_{{KL}}\left(P,U\right)$ denotes the Kullback–Leibler (*KL*) distance between the observed amplitude distribution (*P*) and the uniform distribution (*U*); $H\left(P\right)$ denotes the Shannon entropy (*H*) of a distribution *P*; *f*_*p*_ and *f*_*A*_ denote the two frequency ranges for obtaining the phase and amplitude information; ${\varphi }_{{f}_{p}(t)}$ denotes the phase time series obtained from the Hilbert transform; ${A}_{{f}_{A}(t)}$ denotes the amplitude time series obtained from the Hilbert transform; and ${ < {A}_{{f}_{A}} > }_{{\varphi }_{{f}_{p}(j)}}$ denotes the mean ${A}_{{f}_{A}}$ value at the phase bin *j*. More details of the MI calculation can be found in Tort, et al. ^121^. Here, the frequency range for obtaining the amplitude value was set as 31–90 Hz (gamma band), and the frequency range for obtaining the phase value was swept over a range from 1 to 20 Hz with a step of 0.1 Hz.

### Effects of cognitive load variation on different neural features

To statistically test the effects of cognitive load variation on different neural features, two statistical analyses were conducted: *t*-test and linear mixed modeling.

#### (1) Paired *t*-test and non-parametric cluster-based test

We first conducted two-tailed paired *t*-tests between different task states for each of the eight neural features on each electrode: (1) low-band 1/*f* exponent, (2) low-band 1/*f* offset, (3) high-band 1/*f* exponent, (4) high-band 1/*f* offset, amplitudes of (5) theta (4–7 Hz), (6) alpha (8–12 Hz), (7) beta (13–30 Hz), (8) gamma (31–90 Hz). The effect size Cohen’s *d* = *t*/$\sqrt{n}$ (where *t* is the *t* statistic from the paired *t*-test and *n* is the number of samples, i.e., participants) was then calculated from each electrode for visualization of the scalp map of the effects. Before performing each paired *t*-test, we excluded outliers based on the quantile range method^123^, i.e., the outliers were defined as more than 2 × inter-quartile range (IQR) above the 3rd quartile or below the 1st quartile (implemented by *isoutlier* function in MATLAB).

To address the multiple comparison issues, we performed the non-parametric cluster-based permutation tests^124^ according to the following steps. First, the clusters of significant electrodes were identified from the paired *t*-test as at least four adjacent electrodes with *p* < 0.01. The electrode connectivity map for defining adjacency was based on Supplementary Fig. 6. The *t* values from all significant electrodes in the cluster were summed, denoted as *t*_*sum*_. Second, we created surrogate data by scrambling the condition label of the data by reversing the condition labels in half of the participants (randomly selected for each permutation). After reversing the conditional labels for half of the participants, any conditional effect is expected to disappear. The same *t*-test was then conducted on the surrogate data, and the significant cluster was detected as in the first step. The sum of *t* values from the surrogate data was obtained and denoted as *t*’_sum_. Any cluster detected from the surrogate data is due to chance. This procedure was repeated for 2000 permutations, generating a distribution of *t*’_sum_. The *t*_sum_ obtained from the original data was then compared against the distribution of *t*’_sum_ to calculate the corresponding *p* value of it. Because this procedure turns the entire data space into a single test, it can avoid the multiple comparison issues^124^. In the result, we only show statistically significant clusters of effects based on this non-parametric test.

#### (2) Linear mixed model

The effect observed on a dynamic feature may be an epiphenomenon of another feature. To better examine the inter-dependence of different neural features in their association with cognitive load, we built a linear mixed model (LMM) with the various neural dynamic features concerned serving as independent variables, task state (categorical variable, easy: 0, hard: 1) serving as the dependent variable, and participant serving as random effects in the intercept. All major neural features were involved in the initial model, i.e., low-band 1/*f* exponent and offset parameters (1–25 Hz), high-band 1/*f* exponent and offset parameters (26–90 Hz), theta (4–7 Hz), alpha (8–12 Hz), beta (13–30 Hz), gamma (31–90 Hz), MI (between gamma and 9 Hz at Oz), and gender. Except for MI, all neural features were calculated as averages from electrodes within the corresponding significant clusters obtained from the non-parametric cluster-based test. The LMM model was specified as below and was performed using *lme4*^125^ and *lmerTest*^126^ packages in R^127^. After running the LMM model, we checked the multicollinearity issue based on the variance inflation factor (VIF) method. It was found that the 1/*f* offset parameters have extremely high VIF values (due to their high correlation with 1/*f* exponents) and were thus excluded from the model (see Supplementary Table 5). The beta power also had a VIF slightly higher than 5 and thus was also excluded. All the left independent variables had VIF values lower than 5 (reported in the Results). For the normality test, we applied Shapiro-Wilk’s method. Except for MI, all independent variables that were not normally distributed were transformed to be normally distributed (*p*-values > 0.05 from Shapiro–Wilk test). Although MI still could not pass the normality test after transformation, the normality of its distribution has been largely improved (W statistic from Shapiro-Wilk tests was improved from 0.397 to 0.954 for the easy counting task and was improved from 0.288 to 0.967 for the hard counting task). Specific transformation formulas were reported in the Result section. The final LMM model is as follows:

3 $${task\; state} \sim 	\, 1+{highband\; exponent}+{theta}+{alpha}+{gamma}\\ 	+ {MI}+{gender}+(1{|participant})$$

### Application of machine learning to examine the performance of predicting cognitive load level using different sets of neural dynamic features

To further demonstrate that the integration of different neural dynamic features will show a better performance in predicting cognitive load than using separate features (thus demonstrating the non-redundancy of them), we applied a support vector machine (SVM)-based machine learning approach to classify the data. Because the state data (hard and easy counting) are repeated measures and the individual difference in baseline neural feature value is much larger than the between-condition difference, we applied SVM only to the difference values between the two task states of easy and hard counting. Each participant generated a set of difference values of the neural features. The difference values were calculated as the second counting task minus the first counting task. Because the order was counterbalanced, part of the participants generated easy-minus-hard data samples (labeled as 0), and the rest generated hard-minus-easy data samples (labeled as 1).

The MATLAB function *fitcsvm* was used to implement this machine learning with the following parameter settings: Standardize: true; KernalFunction: gaussian. The outliers were removed before applying machine learning following the above-mentioned procedure. We used a k-fold cross-validation method that equally separated the data into k groups and used one group as validation data and the rest as training data in each iteration. We specified k as 5. Because 152 is not a multiple of 5, there is always one group (randomly drawn) containing 32 samples. Each of the 5 groups will have a chance to play the validation data, i.e., the full circle run through 5 iterations. On top of this, we performed 100 outer iterations and obtained the mean validation accuracy from all obtained results. Since we applied a sufficiently large number of iterations, we did not particularly use a stratified version of k folding. Each data sample contains multiple neural features (more specifically, the difference between the two counting tasks). We selectively used one or multiple features (e.g., alpha, theta, 1/*f* parameters, etc.) to compare the performance of the SVM across different selections.

### Association between neural features and behavioral performance in the hard counting task

Because the high-band 1/*f* exponent is the strongest neural indicator of cognitive load variation, we further tested the association between this neural feature and individuals’ counting performance in the hard counting task. Specifically, an ordinary least squares linear regression model was built with the hard counting performance serving as the dependent variable. We examined the regression results from two different independent variables: (1) the difference in 1/*f* exponents between resting open and hard counting and (2) the difference in 1/*f* exponents between easy and hard counting.

### Statistics and reproducibility

The difference between the two conditions within the same participant group were tested using paired two-tailed *t*-test (Table 1, Fig. 3a, d, and Supplementary Tables 1–3, Fig. 5a). Non-parametric cluster-based test was applied to address multiple comparison issues generated by multiple EEG electrodes (Fig. 3a, d and Supplementary Fig. 5a). The outliers were defined as more than 2 × inter-quartile range (IQR) above the 3rd quartile or below the 1st quartile. The outliers were excluded before performing all statistical tests. The effects of different neural features on the cognitive load were estimated by Linear Mixed Model (LMM). The multicollinearity issue in the LMM was addressed using the variance inflation factor (VIF) method. The classification of cognitive load based on neural features were conducted by applying SVM.

All data were analyzed using customized MATLAB (including EEGLAB plugin), Python, and R scripts. The MATLAB version was R2021a. The EEGLAB version was 2022.0. The Python version was 3.6.6. The R version was 1.2.5019.

### Reporting summary

Further information on research design is available in the Nature Portfolio Reporting Summary linked to this article.

## Supplementary information

Supplementary Information

Description of Additional Supplementary Files

Supplementary Data 1

Reporting Summary

## Data Availability

The anonymized EEG and behavioral data are available upon reasonable request. The source data behind the graphs in the paper are provided in Supplementary Data 1.

## Code availability

The codes used to reproduce the analyses are available at 10.5281/zenodo.8118580^128^.
