# Supplementary material for: Comparative analysis of multifaceted neural effects associated with varying endogenous cognitive load
Source: Commun Biol. 2023 Jul 31;6:795. doi: 10.1038/s42003-023-05168-4 (PMC10390511; doi:10.1038/s42003-023-05168-4)
Supplement: Supplementary file 1 — Supplementary Information [file 42003_2023_5168_MOESM1_ESM.pdf]

Supplementary Information

Supplementary Table 1. Maximum effect sizes (Cohen's d) of paired t-tests between task states for different dynamic features based on female participants.

	1/*f* parameters 1–25 Hz		1/*f* parameters 26–90 Hz		theta 4–7 Hz	alpha 8–12 Hz	beta 13–30 Hz	gamma 31–90 Hz	MI 9 Hz
	exp	offset	exp	offset					
Easy* vs *Hard	0.32	-0.23	**0.74**	**0.75**	-0.30<br>-0.38	0.43<br>0.44	0.46<br>**0.53**	**-0.62**<br>-0.32	-0.32
Open* vs *Easy	-0.36	0.27	0.18	0.23	0.38<br>0.31	0.22<br>0.25	0.34<br>-0.29	0.39<br>-0.12	-0.02
Open* vs *Hard	-0.49	-0.31	**0.77**	**0.81**	-0.37<br>-0.49	**0.52**<br>**0.63**	**0.59**<br>0.42	**-0.62**<br>-0.27	-0.30
Close* vs *Open	**-1.22**	0.44	**1.41**	**1.41**	**0.85**<br>0.42	**1.61**<br>**1.60**	**1.32**<br>**0.70**	**-0.88**<br>**-0.86**	-0.20

** Note: Bold values denote effect sizes larger than 0.5 (medium effect size); exp refers to exponent; MI refers to Modulation Index.*

Supplementary Table 2. Maximum effect sizes (Cohen's d) of paired t-tests between task states for different dynamic features based on male participants.

	1/*f* parameters 1–25 Hz		1/*f* parameters 26–90 Hz		theta 4–7 Hz	alpha 8–12 Hz	beta 13–30 Hz	gamma 31–90 Hz	MI 9 Hz
	exp	offset	exp	offset					
Easy* vs *Hard	-0.27	0.22	**0.61**	**0.57**	**-0.50**<br>**-0.60**	**0.56**<br>**0.58**	0.31<br>0.34	**-0.62**<br>0.36	-0.26
Open* vs *Easy	**-0.52**	0.25	0.41	**0.55**	**0.54**<br>0.34	0.48<br>0.47	**0.58**<br>0.27	0.31<br>-0.37	0.32
Open* vs *Hard	**-0.57**	-0.26	**0.69**	**0.70**	-0.40<br>**-0.55**	**0.70**<br>**0.75**	**0.62**<br>0.40	-0.47<br>-0.36	-0.11
Close* vs *Open	**-0.89**	0.42	**1.35**	**1.30**	**0.75**<br>0.37	**1.56**<br>**1.56**	**1.21**<br>**1.00**	**-0.76**<br>**-0.91**	-0.40

** Note: Bold values denote effect sizes larger than 0.5 (medium effect size); exp refers to exponent; MI refers to Modulation Index.*

Supplementary Table 3. Maximum effect sizes (Cohen's d) of paired *t*-tests between mental states for 1/*f* parameters fitted over 1–30 Hz and over 31–90 Hz.

	1/*f* parameters 1–30 Hz		1/*f* parameters 31–90 Hz	
	exp	offset	exp	offset
Easy* vs *Hard	0.40	0.33	**0.61**	**0.57**
Open* vs *Easy	**-0.50**	-0.24	0.19	0.28
Open* vs *Hard	-0.45	-0.26	**0.65**	**0.67**
Close* vs *Open	**-0.78**	**0.61**	**1.30**	**1.21**

** Note: Bold values denote effect sizes larger than 0.5 (medium effect size); exp refers to exponent.*

Supplementary Table 4. Inter-session reliability of 1/*f* parameters in four mental states (first half versus second half of data).

Elec.	1/*f* exp 1–25 Hz				1/*f* offset 1–25 Hz				1/*f* exp 26–90 Hz				1/*f* offset 26–90 Hz			
	RC	RO	CE	CH	RC	RO	CE	CH	RC	RO	CE	CH	RC	RO	CE	CH
Fp1	.84	.72	.75	.78	.94	.90	.89	.91	.90	.87	.87	.88	.93	.91	.90	.91
Fz	.83	.76	.78	.78	.92	.88	.89	.88	.94	.92	.93	.91	.95	.93	.94	.94
F3	.85	.79	.79	.79	.94	.92	.91	.90	.92	.92	.90	.92	.94	.94	.93	.95
F7	.81	.76	.77	.78	.94	.91	.89	.90	.89	.88	.84	.89	.91	.91	.86	.91
FT9	.83	.69	.78	.76	.92	.88	.88	.90	.86	.86	.82	.88	.89	.89	.84	.90
FC5	.84	.79	.79	.82	.94	.91	.87	.89	.91	.87	.86	.90	.91	.90	.90	.92
FC1	.80	.72	.75	.68	.91	.87	.87	.80	.92	.91	.89	.93	.93	.93	.93	.95
C3	.82	.75	.83	.78	.95	.91	.93	.92	.89	.88	.88	.89	.92	.91	.92	.93
T7	.88	.77	.82	.81	.94	.91	.91	.93	.91	.88	.84	.88	.92	.91	.87	.92
TP9	.81	.80	.84	.84	.93	.91	.91	.87	.94	.89	.89	.90	.92	.89	.87	.90
CP5	.89	.84	.85	.81	.97	.95	.95	.94	.92	.85	.85	.83	.94	.90	.90	.88
CP1	.86	.81	.77	.74	.92	.86	.85	.83	.92	.92	.90	.87	.93	.94	.93	.90
Pz	.86	.74	.81	.73	.89	.82	.87	.82	.95	.93	.92	.92	.96	.94	.94	.94
P3	.88	.87	.82	.83	.94	.94	.89	.90	.94	.91	.91	.91	.95	.93	.92	.93
P7	.86	.82	.78	.82	.96	.92	.88	.88	.92	.86	.87	.86	.93	.89	.88	.87
O1	.87	.81	.81	.84	.95	.88	.86	.84	.95	.90	.91	.90	.94	.89	.89	.91
Oz	.86	.77	.78	.84	.94	.89	.87	.88	.94	.91	.92	.92	.93	.89	.90	.91
O2	.82	.74	.79	.83	.94	.88	.89	.88	.93	.89	.93	.93	.93	.90	.92	.93
P4	.84	.77	.76	.80	.94	.89	.87	.89	.95	.93	.90	.92	.96	.94	.91	.94
P8	.81	.79	.73	.80	.94	.89	.88	.89	.91	.86	.89	.90	.92	.86	.89	.91
TP10	.86	.73	.79	.78	.94	.87	.87	.89	.94	.86	.90	.88	.92	.87	.91	.88
CP6	.87	.83	.82	.85	.95	.92	.92	.93	.90	.91	.89	.88	.93	.93	.91	.91
CP2	.85	.78	.81	.80	.91	.87	.84	.90	.93	.91	.91	.90	.94	.93	.91	.92
Cz	.84	.72	.79	.73	.92	.87	.88	.85	.94	.93	.93	.93	.95	.95	.94	.94
C4	.84	.82	.82	.77	.94	.92	.91	.90	.90	.90	.88	.89	.94	.93	.93	.94
T8	.85	.77	.78	.76	.94	.88	.90	.89	.88	.86	.86	.89	.91	.88	.88	.90
FT10	.85	.71	.74	.74	.90	.84	.87	.85	.87	.83	.80	.86	.89	.85	.85	.90
FC6	.88	.77	.82	.77	.93	.88	.88	.83	.91	.87	.87	.90	.93	.89	.90	.92
FC2	.80	.74	.76	.75	.90	.81	.83	.80	.93	.90	.91	.90	.94	.92	.93	.93
F4	.82	.80	.75	.74	.93	.91	.88	.87	.93	.88	.89	.90	.95	.91	.90	.93
F8	.80	.75	.78	.75	.91	.88	.87	.86	.89	.85	.82	.88	.91	.87	.84	.90
Fp2	.82	.74	.75	.83	.94	.90	.86	.93	.90	.88	.87	.91	.93	.91	.90	.93

** Note: Elec. – electrode; RC – Resting Close; RO – Resting Open; CE – Counting Easy; CH – Counting Hard.*

Supplementary Table 5. Fixed effects estimated using linear mixed model (LMM) and all neural dynamic features.

			CI (95%)			
	b	**SE**	**Low**	**High**	***t***	**VIF**
(Intercept)	5.05	1.44	2.23	7.86	3.51	
gender	-0.03	0.07	-0.16	0.10	-0.45	1.16
high-band 1/f exponent	-4.73	2.78	-10.18	0.73	**-1.70·**	240.35
high-band 1/f offset	2.51	1.67	-0.76	5.78	1.51	273.81
low-band 1/f exponent	1.20	1.37	-1.49	3.89	0.88	15.76
low-band 1/f offset	-2.17	1.15	-4.43	0.09	**-1.88·**	19.65
theta	0.95	0.28	0.41	1.49	**3.43****	4.67
alpha	-0.09	0.12	-0.33	0.15	-0.75	3.91
beta	-0.17	0.55	-1.24	0.91	-0.30	8.67
gamma	-1.58	2.18	-5.85	2.69	-0.73	24.98
MI	1.59	34.08	-65.21	68.40	0.05	1.22

**Note: b: co-efficient in the linear mixed model; SE: standard error; CI: confidence interval; t: t statistics; *** p<.001; ** p<0.01; * p<0.05; · p<0.1; VIF: variance inflation factor. Bold values denote statistical significance at the p < 0.1 level.*

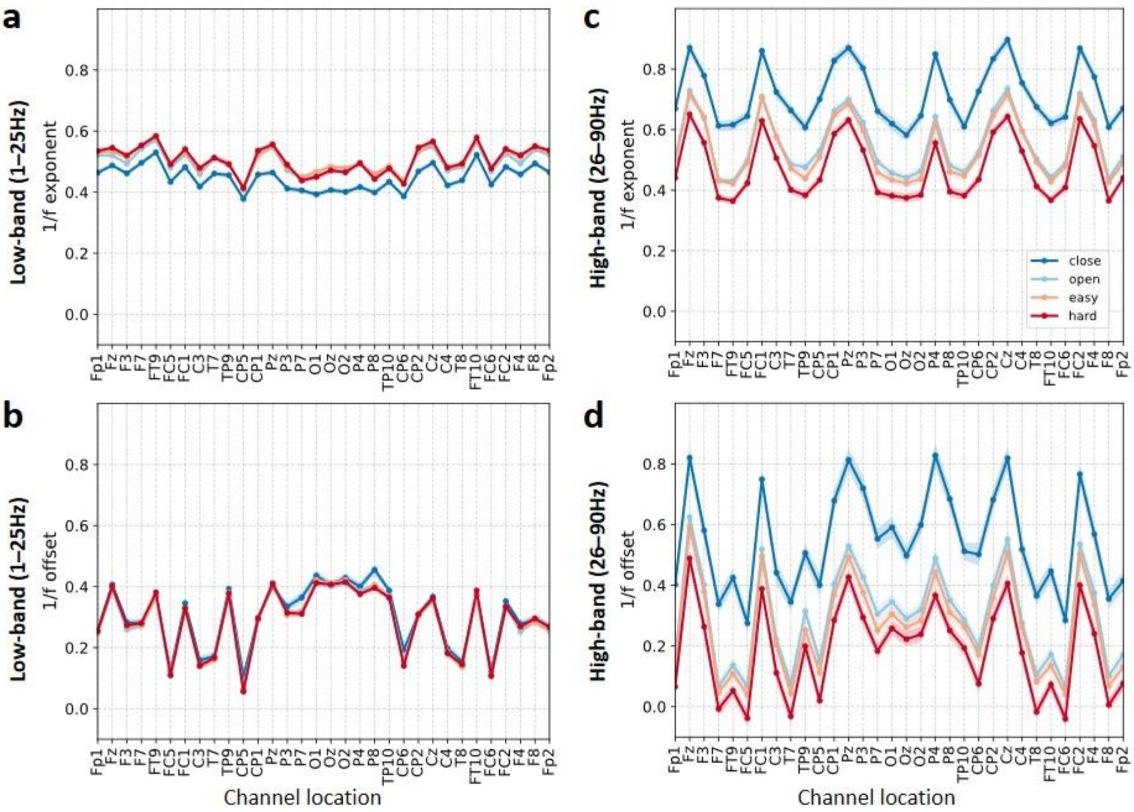

Supplementary Fig. 1 Grand average 1/f parameters fitted using different splitting frequency cutoff. a, b Grand average 1/*f* exponent (a) and offset (b) parameters fitted from 1–25 Hz for different electrodes and states. **c, d** Grand average 1/*f* exponent (c) and offset (d) parameters fitted from 26–90 Hz for different electrodes and states. The standard error across participants was indicated by the background shades behind the lines.

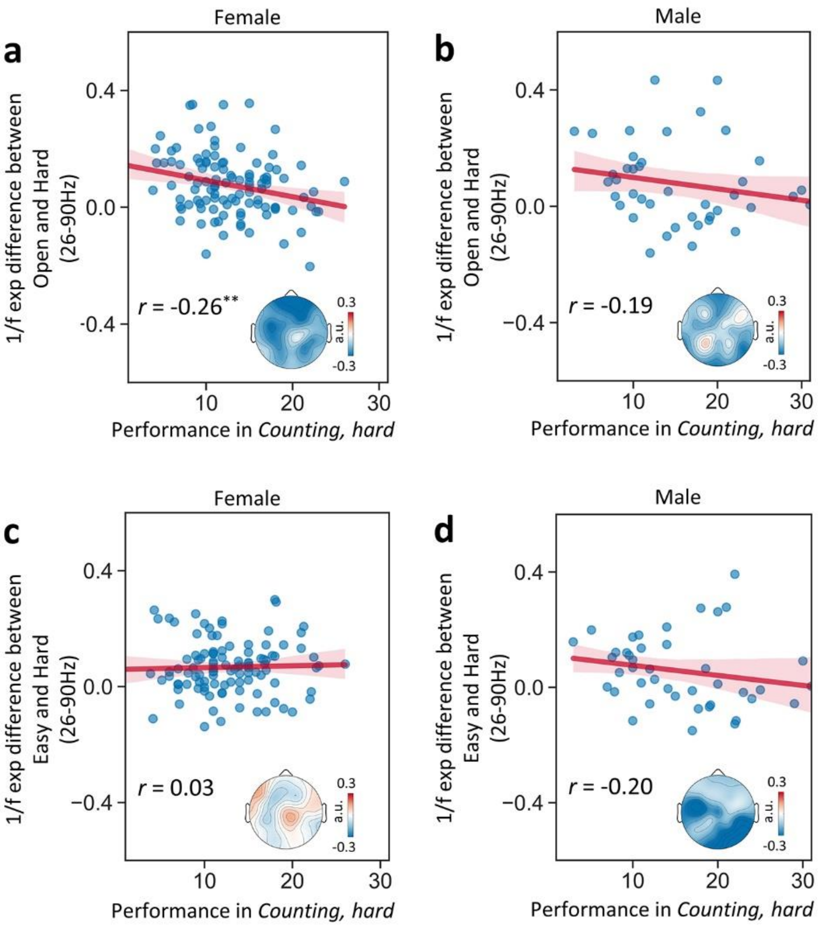

Supplementary Fig. 2 Behavioral association of 1/*f* exponent across genders. a, b Relationship between the behavioral performance in hard counting task and the difference in high-band 1/*f* exponent (fitted from 26-90 Hz, averaged across all electrodes) between resting open and hard counting task in female (a) and male (b) cohort. **c, d** Relationship between the behavioral performance in hard counting task and the difference in high-band 1/*f* exponent (fitted from 26-90 Hz, averaged across all electrodes) between easy and hard counting task in female (c) and male (d) cohort. The 95% confidence interval of the regression line was indicated in red shade. Note: a.u. refers to arbitrary unit.

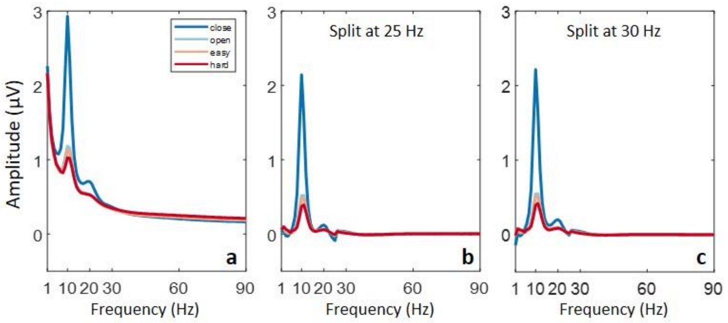

Supplementary Fig. 3 Grand average spectra fitted using different splitting frequency cutoff. a Grand average spectra for the four task states (averaged across all participants and electrodes). **b** Grand average spectra after removing 1/*f* components fitted from 1-25Hz and 26-90Hz. **c** Grand average spectra after removing 1/f components fitted from 1-30Hz and 31-90Hz.

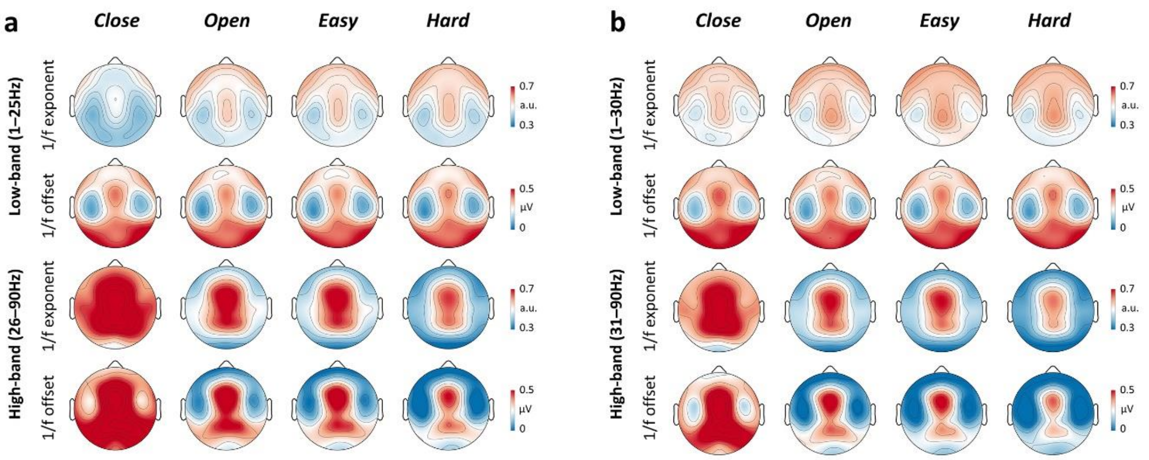

Supplementary Fig. 4 Spatial distribution of the grand average 1/f parameters fitted using different splitting frequency cutoff. a Spatial distribution of the grand average 1/*f* exponent and offset parameters (averaged across all participants) fitted from 1–25 Hz and from 26–90 Hz for each task state. **b** Spatial distribution of the grand average 1/*f* exponent and offset parameters (averaged across all participants) fitted from 1–30 Hz and from 31–90 Hz for each task state. Note: a.u. refers to arbitrary unit.

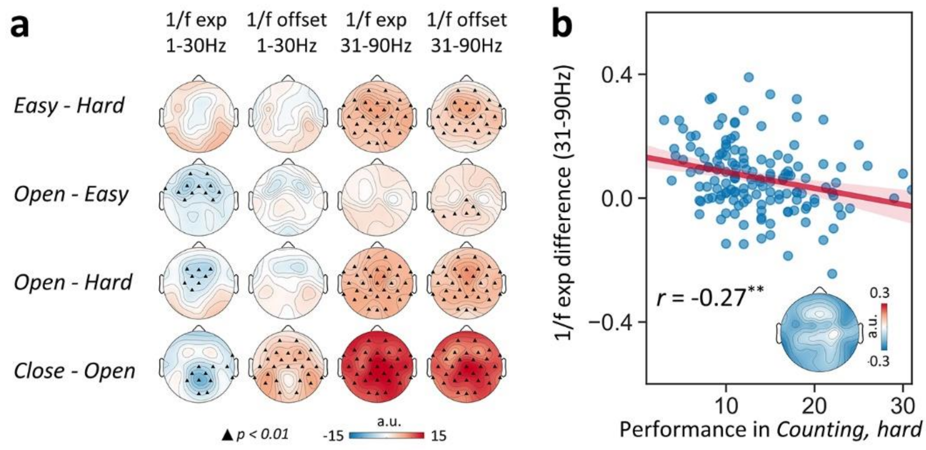

Supplementary Fig. 5 Results of Fig. 3d and Fig. 3b based on 1/*f* parameters fitted from 1–30 Hz and 31–90 Hz. a Topographies of *t* statistics indicating the statistical difference of the various neural dynamic features between different task states. **b** Relationship between the behavioral performance in hard counting task and the difference in high-band 1/*f* exponent (fitted from 31-90 Hz, averaged across all electrodes) between resting open and hard counting task. The 95% confidence interval of the regression line was indicated in red shade. Note: a.u. refers to arbitrary unit.

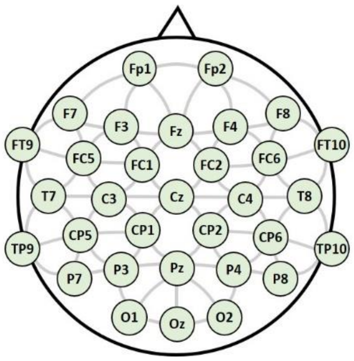

Supplementary Fig. 6 EEG electrode connection defined for calculating significant clusters for paired *t*-tests and cluster-based multiple comparison permutations.
