# Supplementary material for: Comparative analysis of multifaceted neural effects associated with varying endogenous cognitive load
Source: Commun Biol. 2023 Jul 31;6:795. doi: 10.1038/s42003-023-05168-4 (PMC10390511; doi:10.1038/s42003-023-05168-4)
Supplement: Supplementary file 2 — Description of Additional Supplementary Files [file 42003_2023_5168_MOESM2_ESM.pdf]

Description of Additional Supplementary Files

File Name: Supplementary Data 1
Description: Source data underlying main figures.
