# Supplementary material for: Comparative analysis of multifaceted neural effects associated with varying endogenous cognitive load
Source: Commun Biol. 2023 Jul 31;6:795. doi: 10.1038/s42003-023-05168-4 (PMC10390511; doi:10.1038/s42003-023-05168-4)
Supplement: Supplementary file 4 — Reporting Summary [file 42003_2023_5168_MOESM4_ESM.pdf]

nature portfolio

Corresponding author(s): Guang Ouyang

Last updated by author(s): Jul 1, 2023

Reporting Summary

Nature Portfolio wishes to improve the reproducibility of the work that we publish. This form provides structure for consistency and transparency in reporting. For further information on Nature Portfolio policies, see our Editorial Policies and the Editorial Policy Checklist.

Statistics

For all statistical analyses, confirm that the following items are present in the figure legend, table legend, main text, or Methods section.

n/a	Confirmed	
☐	☒	The exact sample size (*n*) for each experimental group/condition, given as a discrete number and unit of measurement
☐	☒	A statement on whether measurements were taken from distinct samples or whether the same sample was measured repeatedly
☐	☒	The statistical test(s) used AND whether they are one- or two-sided<br>*Only common tests should be described solely by name; describe more complex techniques in the Methods section.*
☐	☒	A description of all covariates tested
☐	☒	A description of any assumptions or corrections, such as tests of normality and adjustment for multiple comparisons
☐	☒	A full description of the statistical parameters including central tendency (e.g. means) or other basic estimates (e.g. regression coefficient) AND variation (e.g. standard deviation) or associated estimates of uncertainty (e.g. confidence intervals)
☐	☒	For null hypothesis testing, the test statistic (e.g. *F*, *t*, *r*) with confidence intervals, effect sizes, degrees of freedom and *P* value noted<br>*Give P values as exact values whenever suitable.*
☒	☐	For Bayesian analysis, information on the choice of priors and Markov chain Monte Carlo settings
☒	☐	For hierarchical and complex designs, identification of the appropriate level for tests and full reporting of outcomes
☐	☒	Estimates of effect sizes (e.g. Cohen's *d*, Pearson's *r*), indicating how they were calculated

Our web collection on statistics for biologists contains articles on many of the points above.

Software and code

Policy information about availability of computer code

Data collection	EEG data were collected using BrainAmp DC amplifier (Brain Products, Germany) and stored by BrainVision PyCorder. Behavioral data were recorded by PsychoPy3.
Data analysis	EEG data preprocessing was conducted using MATLAB plugin - EEGLAB 2020.0. 1/f parameters were estimated using FOOOF (python package). LMM analysis was conducted using RStudio and lme4 package. Other statistics analyses and plots were conducted using customized MATLAB codes.

For manuscripts utilizing custom algorithms or software that are central to the research but not yet described in published literature, software must be made available to editors and reviewers. We strongly encourage code deposition in a community repository (e.g. GitHub). See the Nature Portfolio guidelines for submitting code & software for further information.

Data

Policy information about availability of data

All manuscripts must include a data availability statement. This statement should provide the following information, where applicable:

- Accession codes, unique identifiers, or web links for publicly available datasets
- A description of any restrictions on data availability
- For clinical datasets or third party data, please ensure that the statement adheres to our policy

The anonymized EEG and behavioral data in this study are available upon reasonable request.

March 2021

Human research participants

Policy information about studies involving human research participants and Sex and Gender in Research.

Reporting on sex and gender	A total of 156 healthy young adults in Hong Kong completed the experimental tasks in this study. Four participants were excluded due to noncooperative behaviors or technical problems during the experiment. All included participants (42 men and 110 women; age 24.20 ± 3.93 years) reported normal or corrected-to-normal vision and no history of mental disease and gave informed consent.
Population characteristics	see above
Recruitment	The participants were recruited by advertisement.
Ethics oversight	the Human Research Ethics Committee (HREC) of the University of Hong Kong

Note that full information on the approval of the study protocol must also be provided in the manuscript.

Field-specific reporting

Please select the one below that is the best fit for your research. If you are not sure, read the appropriate sections before making your selection.

☒ Life sciences ☐ Behavioural & social sciences ☐ Ecological, evolutionary & environmental sciences

For a reference copy of the document with all sections, see nature.com/documents/nr-reporting-summary-flat.pdf

Life sciences study design

All studies must disclose on these points even when the disclosure is negative.

Sample size	No sample size calculation was performed. The data were obtained from an existing dataset with a total of 152 participants completing all three tasks required for the present study.
Data exclusions	Four participants were excluded due to noncooperative behaviors or technical problems during the experiment.
Replication	Replications were done on subgroups.
Randomization	The order of the three tasks was randomized across participants.
Blinding	not applicable

Reporting for specific materials, systems and methods

We require information from authors about some types of materials, experimental systems and methods used in many studies. Here, indicate whether each material, system or method listed is relevant to your study. If you are not sure if a list item applies to your research, read the appropriate section before selecting a response.

Materials & experimental systems

n/a	Involved in the study	
☒	☐	Antibodies
☒	☐	Eukaryotic cell lines
☒	☐	Palaeontology and archaeology
☒	☐	Animals and other organisms
☒	☐	Clinical data
☒	☐	Dual use research of concern

Methods

n/a	Involved in the study	
☒	☐	ChIP-seq
☒	☐	Flow cytometry
☒	☐	MRI-based neuroimaging
